# Various Simulated Body Fluids Lead to Significant Differences in Collagen Tissue Engineering Scaffolds

**DOI:** 10.3390/ma14164388

**Published:** 2021-08-05

**Authors:** Tomáš Suchý, Martin Bartoš, Radek Sedláček, Monika Šupová, Margit Žaloudková, Gražyna Simha Martynková, René Foltán

**Affiliations:** 1 Department of Composites and Carbon Materials, Institute of Rock Structure and Mechanics, Czech Academy of Sciences, 182 09 Prague 8, Czech Republic; supova@irsm.cas.cz (M.Š.); zaloudkova@irsm.cas.cz (M.Ž.); 2 Faculty of Mechanical Engineering, Czech Technical University in Prague, 160 00 Prague 6, Czech Republic; radek.sedlacek@fs.cvut.cz; 3 Institute of Dental Medicine, First Faculty of Medicine, Charles University and General University Hospital in Prague, 120 00 Prague 2, Czech Republic; martin.bartos@lf1.cuni.cz (M.B.); rene.foltan@vfn.cz (R.F.); 4 Institute of Anatomy, First Faculty of Medicine, Charles University, 120 00 Prague 2, Czech Republic; 5 Nanotechnology Centre, CEET, VŠB-Technical University of Ostrava, 708 00 Ostrava-Poruba, Czech Republic; grazyna.simha@vsb.cz

**Keywords:** simulated body fluid, collagen, blood plasma, mechanical properties, structural parameters, porosity, mass loss, scaffold, micro-CT, XRD

## Abstract

This study aims to point out the main drawback with respect to the design of simulated body environments. Three media commonly used for the simulation of the identical body environment were selected, i.e., Kokubo’s simulated body fluid that simulates the inorganic component of human blood plasma, human blood plasma, and phosphate buffer saline. A comparison was performed of the effects of the media on collagen scaffolds. The mechanical and structural effects of the media were determined via the application of compression mechanical tests, the determination of mass loss, and image and micro-CT analyses. The adsorption of various components from the media was characterized employing energy-dispersive spectrometry. The phase composition of the materials before and after exposure was determined using X-ray diffraction. Infrared spectroscopy was employed for the interpretation of changes in the collagen secondary structure. Major differences in terms of the mechanical properties and mass loss were observed between the three media. Conversely, only minor structural changes were detected. Since no general recommendation exists for selecting the simulated body environment, it is necessary to avoid the simplification of the results and, ideally, to utilize alternative methods to describe the various aspects of degradation processes that occur in the media.

## 1. Introduction

In the course of the development of biomaterials, it is not always possible to perform in vivo animal experiments, especially in the early stages that deal with the determination of the processing parameters and the optimization of the mechanical and structural parameters, evaluation of possible toxicity, etc. The application of experimental animal models is strictly controlled by ethical rules and, most often, it is employed only in the final part of the biomaterial development process. Thus, the in vitro simulation of the body environment represents a useful approach in the overall experimental process. When designing such experiments, the researcher must answer a number of questions: What kind of simulated body fluid or medium to use? What test conditions to apply? What kind of information is required? Is it possible to interpolate the experimental in vitro results to real in vivo conditions or processes? Is it possible to verify them?

Various simulated body fluids formulations are used for more than 30 years to evaluate the ion release, swelling, and biodegradation rates of biomaterials, corrosion resistance, bone-bonding behavior, deposition of calcium phosphates, and last but not least the bioactivity and cytocompatibility of studied materials. One of the most commonly used is simulated body fluid (SBF) proposed by Tadashi Kokubo and Hiroaki Takadama [1]. They originally proposed SBF for the in vitro determination of bone-bonding ability as a replacement for, particularly, the in vivo biological evaluation of ceramic-based materials. SBF and modified versions thereof simulate the ion concentration of the inorganic part of human blood plasma and are commonly suggested in the literature as the medium used for the determination of the degradation profiles of various (not only ceramic) materials [2,3,4,5,6]. SBF enjoys wide popularity in scientific papers; the original research paper [1] contains over 5600 citations (Web of Science Core Collection, 27 July 2021). Simulated body fluids are usually defined as acellular, protein-free, supersaturated calcium-phosphate solutions with ionic compositions almost equal to the composition of human blood plasma generally buffered at physiological conditions (pH = 7.4 and 36.5 °C), e.g., 0.9% sodium chloride aqueous solution, phosphate-buffered saline (PBS) [7,8], Hank’s balanced salt solution (HBSS), etc. [9]. However, some simulated body fluids, e.g., conventional SBF and Dulbecco’s Modified Eagle Medium (DMEM) also contain artificial buffers that are not present in blood plasma such as TRIS (tris(hydroxymethyl)aminomethane) and HEPES (N-(2-hydroxyethyl)piperazine-N′-(2-ethanesulfonic acid)), respectively [10]. However, accurate in vivo simulation requires simulated body fluids that are modified by molecules of biological origin such as amino acid [11], proteins [12,13], glucose, vitamins [14], and enzymes [15,16]. The basis of the Minimal Essential Medium (MEM) [17] consists of six salts and glucose supplemented with 13 essential amino acids and eight vitamins. Other MEM variations such as DMEM [17] or α-MEM [17] have been developed mostly by adding additional vitamins, amino acids, and/or other nutrients [18]. In addition, for the purposes of in vitro cellular testing, such media are further supplemented with bovine serum albumin and antibiotics. The most recent detailed overview of the various simulated body fluids used in biomaterial engineering is presented in Yilmaz et al. [19].

Media composed of inorganic ions and organic molecules form complicated systems in which a number of mutual interactions occur between the studied material and the components in the medium [20]. In addition, if the studied material is degradable, this allows for a variety of other reactions. It has been proved that organic molecules are capable of providing the in situ biomimetic precipitation of calcium phosphates (CaPs). Šupová et al. [21] determined that the ratio of the ionic/non-ionic forms of calcium in pure DMEM was altered particularly in the presence of CaP nanopowders. Simultaneous precipitate/dissolution processes are capable of inducing changes in pH values, which may influence the adjustment of equilibria in the media and the solubility and, consequently, the stability of the precipitates. Both biomimetic precipitation and massive ion uptake from the medium are able to strongly influence the mechanical properties [22] and cell viability [23], respectively.

Since simulated body fluids play a critical role in the aforementioned processes, the compositional aspects of such media must be seriously considered, since they exert significant impacts on the mutual interactions that occur in the media. For example, Zhao et al. [24] determined that the presence of bovine serum albumin strongly inhibited the formation of HA in traditional SBF, while HA could still be observed in carbonate-buffered SBF. However, some molecular simulations attempted to visualize a collagen–CaP hybrid in pure water via the use of calcium, phosphate, and hydroxy ions only, without considering the roles of other biologically relevant molecules [25]. There is no limit relating to the composition of versions of SBF solutions, and they can be applied in many different in vitro tests for specific purposes. However, due to the high level of variability concerning the composition of the various media, it is practically impossible to compare the same processes that occur in different media and under differing operational procedures. Therefore, discrepancies may occur between the final conclusions of different researchers in the field. Nevertheless, the immersion of samples in SBF remains the most widely applied, simple, and economical method for the assessment of the properties of biomaterials.

The selection of simulated body conditions may reflect the specific requirements of the application of scaffolds. For example, in a previous study by Suchý et al. [26], the authors employed a cell cultivation medium for simulating in vitro biological evaluation conditions by means of mesenchymal stem cells. Hence, the same medium and conditions, e.g., temperature and atmosphere, were applied. The results consisted of the prediction of the mechanical and structural behavior during the in vitro test. Such information is beneficial in terms of the interpretation of cell behavior, since changes in both the mechanical and structural properties of scaffolds may exert an important influence in this respect.

The aim of this study is to illustrate the importance of the selection of in vitro simulated body conditions via a simple experiment involving the comparison of the effects of three different media that are used for the simulation of the identical body environment and commonly reported in the literature on the mechanical and structural properties of collagen scaffolds. The study involved the preparation of collagen scaffolds based on collagen type I due to it comprising the most common material employed in the preparation of tissue engineering constructs. Three different media were used for the simulation of in vivo conditions, the first of which was an SBF proposed by Kokubo and Takadama [1], human blood plasma, and phosphate buffer saline (PBS). PBS is one of the most commonly used media with respect to degradation experiments due to its widespread availability, simplicity, and physiological pH. The mechanical and structural effects of each medium were determined via the conducting of compression tests, the determination of mass loss, and image and micro-CT analysis. Finally, infrared spectroscopy was employed for the interpretation of possible changes in the secondary structure of the collagen. These methods were selected as representative analysis methods published with concern to similar experiments that assessed the impact of simulated body conditions. The design of this study was significantly simplified. The effects of enzymatic degradation, inflammatory reactions, dynamic conditions, and cellular activities were omitted so as to allow for the simulation of similar conditions commonly published in the literature.

## 2. Materials and Methods

### 2.1. Collagen Scaffolds

Collagen type I isolated from calf skin (VUP Medical, Brno, Czech Republic) was used for the preparation of the scaffolds. The collagen was allowed to swell in deionized water for 1 h at a temperature of 20 °C, homogenized using a disintegrator (10,000 r.p.m., 10 min), and left for 60 min at a temperature of 20 °C. Following this delay, the dispersion was further homogenized (10,000 r.p.m., 1 min) and placed in cylindrical containers with diameters of 12 mm or 6 mm. The final concentration of the aqueous collagen dispersion was 4 wt%. The containers holding the collagen dispersion were frozen at −80 °C for 3 h and then lyophilized (BenchTop 4KZL, VirTis, Los Angeles, CA, USA). The cross-linking of the collagen scaffolds was performed using N-(3-dimethylamino propyl)-N′-ethylcarbodiimide hydrochloride (EDC) and N-hydroxysuccinimide (NHS) at an EDC/NHS ratio of 4/1 (wt/wt). The EDC and NHS were dissolved in a 95 wt% ethanol solution. The EDC and NHS were purchased from Sigma-Aldrich, St. Louis, MI, USA. The scaffolds were exposed to the cross-linking solution for 24 h at a temperature of 37 °C. Then, 0.1 M Na_2_HPO_4_ (45 min) was used for the double washing of the scaffolds. The final preparation stages comprised rinsing in deionized water (30 min), freezing (−80 °C), and lyophilization.

### 2.2. Media Exposure

The SBF was prepared in accordance with the original protocol published by Kokubo [1]. With respect to the comparison of the influence of SBF, human blood plasma (3 donors with different blood groups, sex, and age) supplemented with antibiotics (penicillin (20 mg mL^−1^; Sigma-Aldrich, St. Louis, MI, USA), and streptomycin (20 mg mL^−1^; Sigma-Aldrich, St. Louis, MI, USA) was employed as the second medium. The plasma was separated from the whole blood by means of standard procedures defined in the Guide to the Preparation, Use, and Quality Assurance of Blood Components (Recommendation No. R (95) 15, European Directorate for the Quality of Medicines & Health Care) and legislation valid in the Czech Republic (Decree 143/2008 Collection of Laws). Briefly, the plasma was separated from the blood and collected in blood bags with integral transfer packs employing hard-spin centrifugation with freezing (temperature below −25 °C) that commenced within 6 h. The diagnostic samples obtained from the donors were examined applying standard practice for signs of infection (HIV, HBV, HCV, syphilis). The requirements for the composition of the plasma were also verified, i.e., factor VIII (not less than 70 IU per 100 mL), residual cells (red cells < 6 × 10^9^/L, leucocytes < 0.1 × 10^9^/L, platelets < 50 × 10^9^/L), and the whole content of protein (at least 50 g/L). Finally, phosphate buffer saline (PBS; Sigma Aldrich, St. Louis, MI, USA) was selected as the third medium. In all three cases, the samples were placed in separate 30 mL test tubes with a weight/volume ratio of 26.7 ± 3.9 mg/25 mL of each medium. The test tubes were tightly sealed; thus, the amount of the media during exposure could safely be considered to be the same. The samples were incubated at 37 °C in a normal atmosphere for up to 14 days.

### 2.3. Compression Test

The mechanical behavior of the scaffolds in the hydrated state (10 min of soaking) and 7 and 14 days following exposure in the media was determined by means of the conducting of compression tests that adopted the ISO 13314 standard (the compression test for porous and cellular metallic materials). Ten samples (*n* = 10) with lengths of 14 mm and diameters of 12 mm were tested for each group and time point. The MTS Mini Bionix 858.02 system (MTS, Eden Prairie, MN, USA) was used for the determination of the plateau stress and elastic gradient. In the case of porous materials, the plateau stress represents the compression strength, and the elastic gradient represents the modulus of elasticity under compressive loading as the closest concept to that of solid materials [27]. Here, 10 N and 100 N load cells were used. A constant crosshead speed of 3.0 mm min^−1^ was applied for the measurements (deformation rate ≈ 5.0 × 10 ^−3^ s^−1^). The arithmetical mean of the stresses between 20% and 30% of the compressive strain was used for the calculation of the plateau stress. The calculation of the elastic gradient was performed via elastic loading and unloading between stresses of 70% and 20% of the plateau stress and the determination of the gradient of the elastic straight lines.

### 2.4. Mass Loss

Seven and 14 days following exposure in the media, the mass losses were calculated for the determination of the in vitro degradation rate of the scaffolds. Scaffolds from each of the groups (*n* = 8) were removed from the media, washed with deionized water, and lyophilized following freezing at −30 °C (for 5 h). The mass loss was calculated via the following equation: *mass loss* = (*W_0_ − W_t_*)/*W_0_*, employing the initial dried weight of the sample (*W_0_*) and the dried weight of the sample following degradation (*W_t_*).

### 2.5. Scanning Electron Microscopy and Energy-Dispersive Spectrometry

A Quanta 450 electron microscope (FEI, Hillsboro, OR, USA) was used for the scanning electron microscopy (SEM) of the scaffold in the high vacuum mode. An ion sputter (Emitech K550X, Quorum Technologies, Kent, UK) was used for the coating of the samples with a thin layer of gold. The adsorption of various components from the blood plasma, SBF, and PBS following exposure was qualitatively characterized via energy-dispersive spectrometry (EDS) using a Quanta 450 scanning electron microscope (FEI, Hillsboro, OR, USA) equipped with an Apollo XL Silicon Drift Detector 1 EA EDS extension (EDAX Genesis system, Mahwah, NJ, USA) at a magnification of 1000× and an accelerating voltage of 12.5 kV. Prior to the EDS analysis, dried samples (*n* = 5) were sputter-coated with carbon in an ion sputter (Emitech K550X, Quorum Technologies, Kent, UK). The weight fractions of the chemical elements incorporated within the dried samples before and after exposure in the media were characterized.

### 2.6. Micro-CT Analysis

The collagen scaffolds (*n* = 20) were micro-CT scanned using a SkyScan 1272 desktop ex vivo device (Bruker micro-CT, Kontich, Belgium) with the following parameters: pixel size 4 μm, source voltage 60 kV, source current 166 μA, no filter, rotation step = 0.2°, frame averaging (2), rotation 180°, scanning time approximately 1 h. The same specimens were scanned once more following exposure to the relevant medium (SBF, PBS, blood plasma) and re-lyophilized. The total number of scanned specimens was 40. The specimens were mounted on specimen holders and scanned in the dry state in air. Projection images were reconstructed using NRecon software (v.2.8.0., Bruker, Kontich, Belgium). Visualizations were acquired using a DataViewer (v.1.5.2.4, Bruker, Kontich, Belgium) and a CTVox (v.1.5, Bruker, Kontich, Belgium). The volume of interest (VOI) was set inside the specimen so as to exclude those superficial parts which may have been altered via the handling of the specimens. The dimensions of the VOI were the same in all the specimens. The image processing (noise reduction, filtration, and despeckle operations) and binarization were conducted in CTAn software (v.1.18, Bruker, Kontich, Belgium) and optimized using TeiGen software [28]. The structure analysis was performed using 3D analysis in CTAn. The pore size evaluation was performed using a sphere-fitting algorithm.

### 2.7. Infrared Spectroscopy

The infrared spectra were measured using an iS50 spectrometer (Thermo Nicolet Instruments Co., Madison, WI, USA) over a range between 4000 and 400 cm^−1^ at a resolution of 4 cm^−1^, averaging 64 scans via the ATR technique. The spectral deconvolution of the amide I band was performed by means of the OMNIC 7 program (Thermo Nicolet Instruments Co., Madison, WI, USA). The starting parameters for the curve fitting process, i.e., the number bands and their positions, were predetermined by applying the combined procedures of the secondary derivative method and Fourier self-deconvolution. The peak type was defined as Gaussian with an FWHH = 0.964 (FWHH defines the narrowest peak determined by the automatic peak-finding routine). The sensitivity parameter (which determines the polynomial order used in the calculation) was set at high (polynomial order = 6), and the baseline was selected as linear. The fitting procedure automatically adjusted the peak center, height, and width so as to produce a composite spectrum that matched the original. All the materials were measured at 10 different locations. The integral absorbances of the individual spectral components were statistically evaluated.

### 2.8. X-ray Diffraction Analysis

The X-ray diffraction (XRD) patterns of the collagen samples were obtained using an Ultima IV Rigaku X-ray diffractometer, reflexion mode, working conditions Cu Kα (λ = 0.15406 nm) radiation—40 kV, 40 mA; K-beta filter; CBO selection slit—BB; scintillation counter; continuous scan; scan speed/duration time 1°/min; step width—0.02°; scan axis -2Θ/; scan range—5–60° 2Θ; incident and receiving slit 1–2/3°; receiving slit 2–0.6 mm. The International Centre for Diffraction Data (ICDD) database powder diffraction file (PDF)-4+ 2019 was used for the phase analysis. The diffraction data was drawn using Origin 9.1. software (9.6.5.169, OriginLab, Northampton, MA, USA).

### 2.9. Statistical Evaluation

Statistical software (STATGRAPHICS Centurion XVII, StatPoint, Warrenton, VA, USA) was used for the statistical evaluation of all the data. Firstly, the test assumptions were verified by means of the Shapiro–Wilk test (the verification of normality) and the Levene test (the verification of homoscedasticity). Due to the violation of one of these assumptions, non-parametric procedures were employed, i.e., the Kruskal–Wallis multiple sample comparison with post hoc tests (Bonferroni procedure). The Mann–Whitney U-test was performed in the case of two-sample comparisons. The parametric multiple sample comparison was performed using the Games–Howell test. Statistical significance was accepted at *p* ≤ 0.05.

## 3. Results

### 3.1. Compression Test

**Figure 1:**
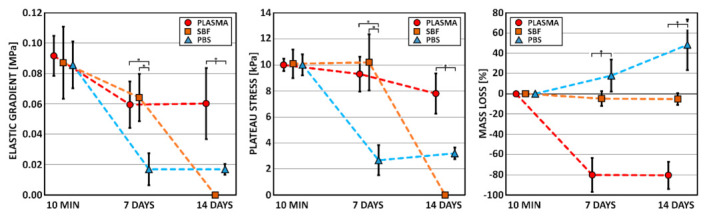
Elastic gradient (elastic modulus), plateau stress (compressive strength), and mass loss of the collagen scaffolds immersed in human blood plasma, SBF, and PBS for 10 min, 7 days, and 14 days (arithmetical mean with standard deviation, *n* = 10). * denotes statistically significant differences (Games–Howell, *0.05*).

Although each of the three media used in this study simulated the same human body environment, the behavior of the collagen scaffolds differed significantly. The differences in the mechanical behavior of the collagen samples are illustrated in Figure 1 (left, middle) and in Figure S1 in the Supplementary File (stress–strain curves). The principal differences can be observed in the case of the human blood plasma and SBF, which simulated its inorganic part ion concentration. Although the mechanical behavior of the scaffolds remained comparable within the first 7 days, 14 days following immersion in the SBF, all the samples had completely degraded with regard both to the elastic gradient and plateau stress. Surprisingly, the vigorous loss of mechanical stability was not accompanied by a measurable mass loss.

### 3.2. Mass Loss

The differences in the degradation of the collagen samples are illustrated in Figure 1 (right). The scaffolds immersed in SBF evinced only a slight increment in weight, while the samples immersed in plasma evinced an approximately 80% increment in weight. Conversely, the scaffolds immersed in PBS evinced an approximately 50% decrease in weight. This illustrates the problematic nature of the determination of the degradation profile of such highly porous and hydrophilic materials. The increases in weight (negative mass loss) can be explained by the precipitation of salts from the SBF and by the adsorption of various components (proteins, saccharides, and vitamins) of the blood plasma following exposure.

### 3.3. Scanning Electron Microscopy and Energy-Dispersive Spectrometry

**Figure 2:**
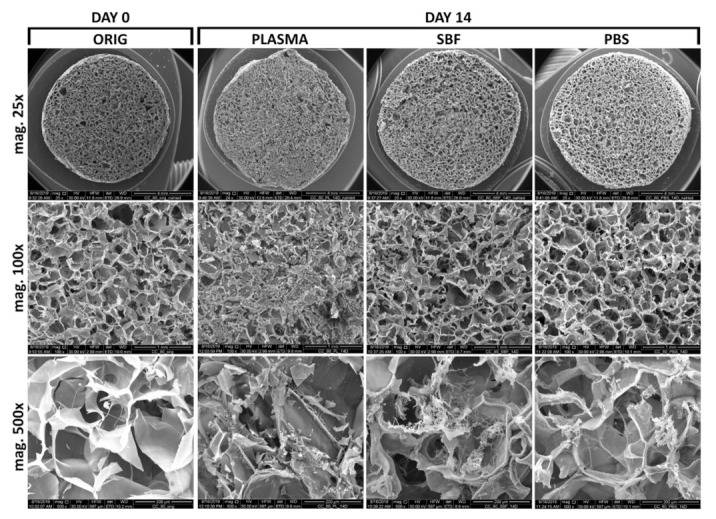
SEM images of the original collagen scaffolds and those immersed in human blood plasma, SBF, and PBS for 14 days.

**Figure 3:**
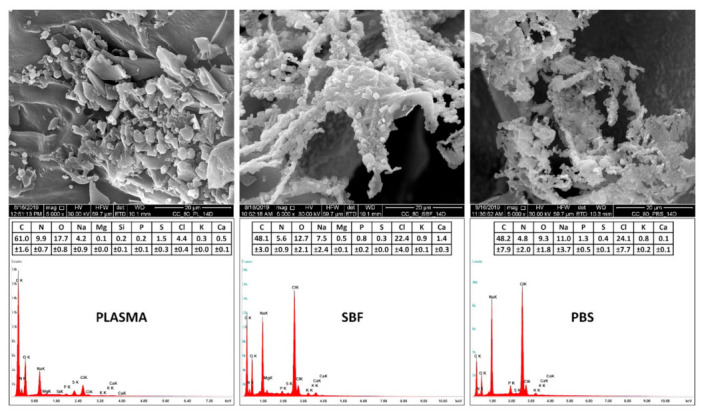
Representative SEM images (mag. 5000×) of the inner structure of collagen scaffolds exposed for 14 days in human blood plasma, SBF, and PBS. The detailed images reveal the adsorption of various components of the blood plasma and salt precipitates after exposure in selected media. The adsorption of the blood plasma components and salts is illustrated by the qualitative EDS analysis (mean and standard deviation, *n* = 5), namely by qualitative increases in weight fraction (wt%) of the Mg, Si, P, Cl, K, and Ca elements. The inner structure of collagen scaffolds prior to experimentation (data not shown) contained only C (62.9 wt%), N (13.7 wt%), O (20.8 wt%), Na (0.5 wt%), S (0.7 wt%) and Cl (1.4 wt%) elements.

The effect of the three various media on the inner structure of the collagen scaffolds is illustrated by the SEM micrographs in Figure 2 and Figure 3. The SEM images and the qualitative EDS analysis (Figure 3) reveal the adsorption of the various components of the blood plasma (e.g., proteins, saccharides, vitamins, etc.) and salt precipitates following exposure in the selected media, all of which contributed to the assessed mass increase in the case of the blood plasma and SBF.

### 3.4. Micro-CT Analysis

**Figure 4:**
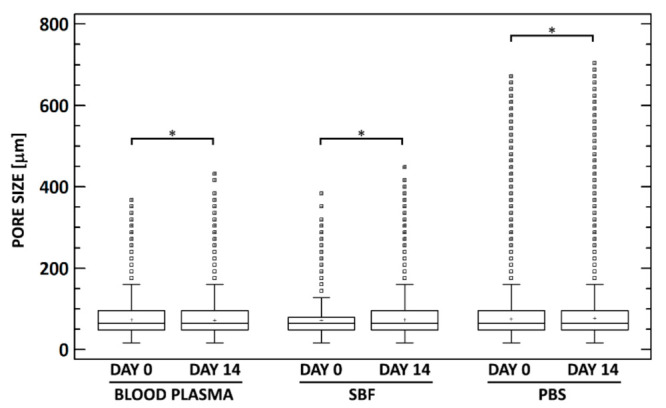
Comparison of the pore size values for all three groups prior to and following exposure. Only minor differences can be observed in terms of porosity volume, pore size, and pore distribution. * denotes statistically significant differences (Mann–Whitney, *n* = 20, *0.05*).

**Figure 5:**
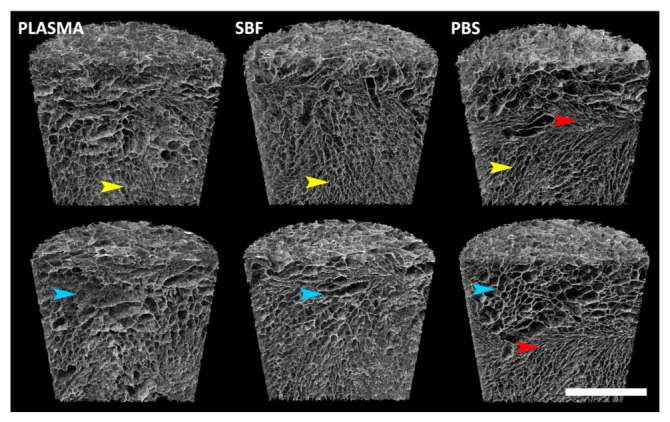
Micro-CT 3D visualizations of representative specimens from each group. The upper images were taken prior to media exposure, while the lower images show the same specimens following media exposure. No apparent structural changes occurred. The structure is not strictly homogeneous. Various areas in each of the specimens are highlighted: small pores (yellow), large pores (blue), condensed area (red). Scale bar = 2 mm.

Micro-CT 2D (cross-section images) and 3D visualizations were prepared and examined. The scaffolds were generally highly porous, and their structure usually consisted of areas with higher and lower pore sizes and condensed structures (Figure 4 and Figure 5). No apparent structural differences resulted from exposure to the media. In order to verify these observations, the scaffolds were subjected to 3D analysis (Table 1). Only minor changes were observed in the specimens following cultivation: a ≈ 2–3% increase in the volume of the scaffolds, a ≈ 3% increase in the surface of the scaffolds, a 1 µm increase in the wall thicknesses of the specimens, and a ≈ 2–3% decrease in the porosity of the scaffolds (Table 1). The closed porosity was below 0.01% for all the specimens, thus indicating highly interconnected pores. The differences in the structural parameters between the groups with the various cultivation media exposures were lower than 1.2% with concern to all the parameters.

### 3.5. Infrared Spectroscopy

Since infrared spectroscopy (FTIR) can be used as an effective analytical tool for the interpretation of the secondary structure of collagen and its structural changes following various processes, this method was applied for the evaluation of the secondary structure of the collagen following immersion in the media.

The FTIR spectra of collagen contain five amidic bands (A, B, I, II, III). The peak, centered at 3330 cm^−1^, comprises a mutual band of hydrogen bonds from the intermolecular water and amide A of collagen associated with N-H stretching. The band at ≈3080 cm^−1^, quoted as amide B, is a common band for C–H stretching in the sp_2_ hybridization and stretching vibration of the N-H bonds in secondary amides. Generally, amide I bands (≈1650 cm^−1^) originate from C=O stretching vibrations coupled with N–H bending vibrations. Amide II bands (≈1550 cm^−1^) arise from N–H bending vibrations coupled with C–N stretching vibrations [29,30]. Other proofs of the existence of the triple helical structure consisted of the presence of a quartet of bands at ≈ 1205, 1240, 1280 (amide III), and 1340 cm^−1^ [31,32].

**Figure 6:**
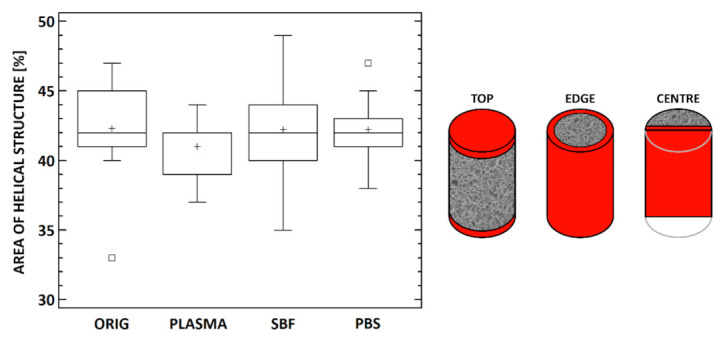
**Left**: The area of the helical collagen structure determined via the FTIR analysis for the samples prior to exposure (ORIG) and following exposure to the media: blood plasma (PLASMA), simulated body fluid (SBF), and phosphate buffer saline (PBS). No statistically significant differences were detected (Kruskal–Wallis test with the Bonferroni procedure, *0.05*, *n* = 10). **Right**: Scheme of the positions as evaluated via FTIR. The thickness of layer evaluated by FTIR is up to approximately 10 μm.

The amide I region of the collagen spectrum (≈1650 cm^−1^) can be deconvoluted into several distinct bands with maxima at ≈1690–1670, 1660, 1650, 1630–1635, and 1610–1615 cm^−1^ [33]. The band at ≈1660 cm^−1^ was assigned to the triple helix, with a contribution from the α-helix, while the band at ≈1630 cm^−1^ was assigned to the aggregate β-sheet structure (left-handed 3-10 helix in the denaturated state) [33]. However, the spectra also contained other components that corresponded to other structural states. The component at ≈1615 cm^−1^ related to gelatin [30], and the band at ≈1650 cm^−1^ corresponded to random coils and imide residues [33]. The band at ≈1670–1690 cm^−1^ was attributed to the β-turn and helices of aggregated collagen-like peptide (the antiparallel β-sheet structure) [33]. The deconvoluted spectra of all the studied materials are presented in the Supplementary file (Figures S2–S5). The deconvolution of the amide I region revealed four bands; i.e., at ≈ 1685, 1660, 1633, and 1613 cm^−1^; no band 1650 characteristics for random coils were revealed in any of the materials due to cross-linking. The determination of part of the triple helical structure represented by the band at 1660 cm^−1^ following immersion in the various media was statistically evaluated (Figure 6, left). No statistically significant differences were detected relating to the area of the band at 1660 cm^−1^ that represented the helical part between the collagen materials immersed in the various media.

**Figure 7:**
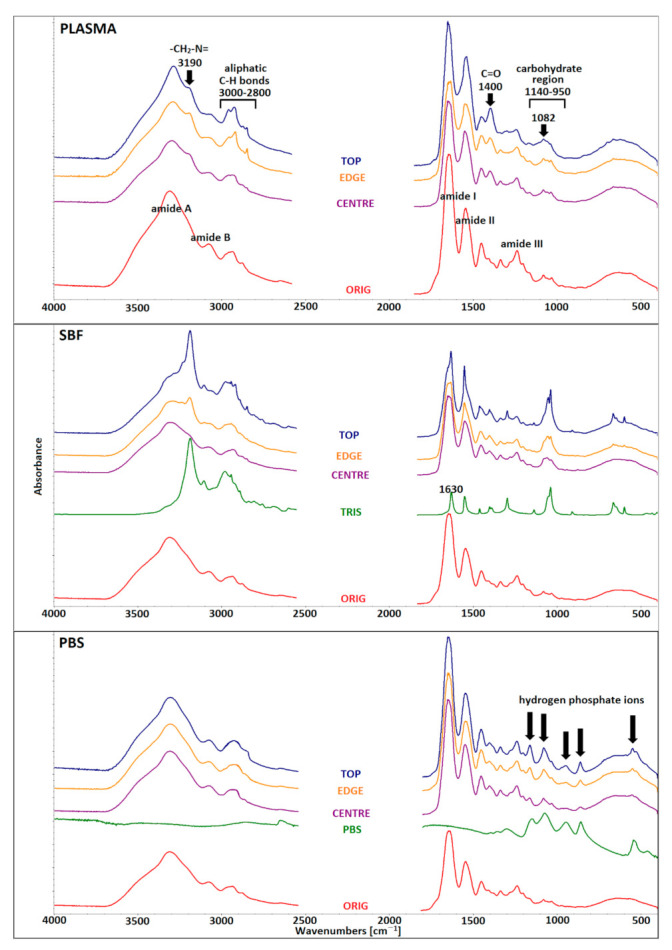
Comparison of the FTIR spectra of all the studied materials. The middle-infrared spectral region (4000–400 cm^−1^) was employed for comparison purposes.

All the media used contained components such as inorganic ions or organic molecules. The PBS contained inorganic salts such as disodium hydrogen phosphate, sodium chloride and, in some formulations, potassium chloride, while the SBF contained, in addition to inorganic ions such as hydrogen phosphate and hydrogen carbonate, TRIS hydrochloride (tris(hydroxymethyl)aminomethane hydrochloride) organic buffer. The blood plasma comprised a multicomponent system that contained proteins (albumins, globulins, and fibrinogen), glucose, hormones, clotting factors, and electrolytes. In order to evaluate the homogeneity of the penetration of the various components into the internal structure of the materials, the samples were measured in three different positions, i.e., in the center, on the top, and on the edge of the scaffold (Figure 6, right). The proteins in the blood plasma, as well as the amino group in the TRIS contained in the SBF, were capable of spectrally interfering with the amide I band. Human serum albumin, the most abundant protein in plasma, embodies predominantly an α-helical structure and, upon interaction with neutral salts, may lead to the formation of intermolecular β-sheets [34]. Both these secondary structures are able to influence the deconvoluted band areas in amide I. The alpha-helix in native human albumin adopts band 1650–1660 cm^−1^, while intermolecular β-sheets can be detected in the spectral range 1610–1630 cm^−1^ [35]. The amino group in TRIS adopts in-plane bending deformation at 1630 cm^−1^ (Figure 7 SBF), which is typical for primary amines. Since these components could spectrally interfere with the amide I band, only the areas from the middle part (CENTER), which were least influenced by these components, were employed with respect to both the deconvolution of the amide I band and the further determination of the percentage of the helical component of the collagen.

As can be seen from Figure 7, in the cases of the SBF and the blood plasma, the middle part of the scaffold (CENTRE) was least affected by the components. However, this effect is more obvious in the peripheral parts of the scaffold (EDGE and TOP). Bands of TRIS comprised the most significant spectral component in the SBF. In the case of the blood plasma, a slight increase in the absorbances at 3190 cm^−1^ (asymmetric -CH_2_-N= in the Pro and Hyp side chains [33]) and 2800–2950 cm^−1^ (C-H aliphatic bonds), and increases in the band at ≈1400 cm^−1^ (C=O in the carboxyls and esters) and in the carbohydrate region (950–1140 cm^−1^) are visible predominantly in the TOP part. It has been proposed that the carbohydrate region comprises a proteoglycan content marker and is composed of seven underlying bands. The sub bands (995 cm^−1^, 1012 cm^−1^, 1049 cm^−1^, 1064 cm^−1^, and 1101 cm^−1^) were reduced following trypsin treatment, which suggested that these sub bands were generated by non-collagenous proteins [36]. The 1064 cm^−1^ band has been reported as a sulfated proteoglycan marker in cartilage [37]. The 1082 cm^−1^ sub band can be assigned to the mutual stretching vibration of the C-O bonds in the carbohydrate [38] as well as to the symmetric PO_2_ stretching vibration in the phosphorylated proteins [31]. The intensity ratio of the 1032 cm^−1^ peak to the amide I peak increased following collagen glycation, which supports the use of the 1032 cm^−1^ band as a collagen glycation marker [39]. Glucose comprises one of the most significant carbohydrates in blood plasma. The changes in the spectra mentioned above most probably correlate with the adsorption of some of the components of the blood plasma. However, due to its multicomponent composition and low concentration of adsorbed components, this is difficult to identify precisely. In the case of the PBS, the most striking spectral component comprises hydrogen phosphate anions, which are also slightly apparent in the middle part of the scaffold (CENTRE); however, they do not interfere with the amide I band.

### 3.6. X-ray Diffraction

**Figure 8:**
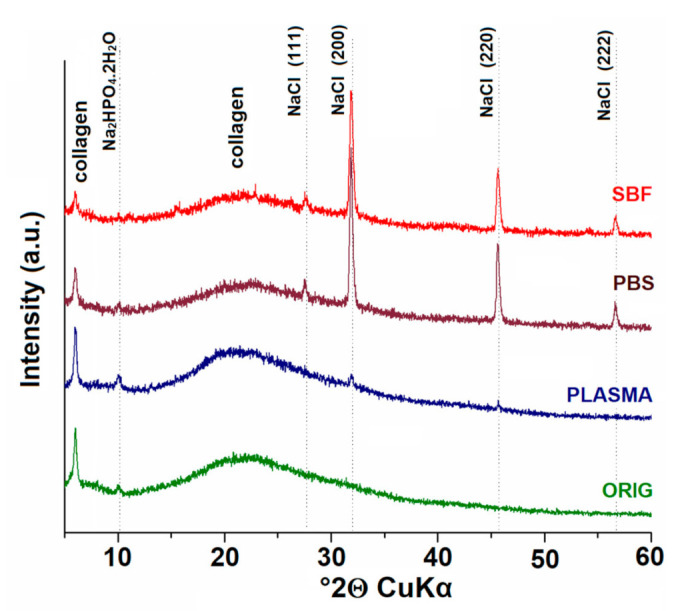
Comparison of the XRD patterns of the original collagen (ORIG) and the collagen exposed to all the studied media (PLASMA, PBS, and SBF).

The XRD method allowed for the determination of the phase composition of the studied materials. The XRD patterns of all the materials are shown in Figure 8. The XRD patterns of all the studied materials evinced two diffraction peaks at ≈6° and ≈20° 2Θ. The first diffraction peak was relatively sharp and the second was broad, which is consistent with the characteristic diffraction peaks of collagen [40]. The first diffraction peak in the region 6–7° 2Θ reflected the distance between the molecular chains of the collagen fibrils, which varied from 1.2 to 1.5 nm depending on the humidity [41]. The second diffraction peak of around 20° 2Θ reflected the diffuse scatter caused by the large number of structural layers of the collagen fibrils. A further phase corresponding to a peak at ≈10° 2Θ, although of low concentration, comprised Na_2_HPO_4_ 2H_2_O (dorfmanite, ICDD, PDF card no. 00-010-0190), which was obvious in the XRD pattern of the original collagen (ORIG) and in the patterns of the collagen exposed to blood plasma (PLASMA) and PBS. The other series of reflections (at ≈27°, ≈32°, ≈46°, and 57° 2Θ) confirmed the presence of NaCl (halite, ICDD, PDF card no. 01-072-1668). Although NaCl was present in high concentrations in the patterns of the collagen exposed to both SBF and PBS, the content of NaCl was relatively low with respect to the collagen exposed to blood plasma.

## 4. Discussion

Although the SBF simulated the blood plasma ion concentration, the ionic species of the concentrated solution altered the collagen solubility and influenced its mechanical properties. With respect to both the mechanical properties and the degradation processes, the results obtained were mutually inconsistent. It can be hypothesized that the individual application of SBF would lead to the conclusion that collagen scaffolds degrade in the human body within the first 14 days and that this process is accompanied by a total loss of their mechanical stability. Conversely, the individual application of human blood plasma would lead to the conclusion that collagen scaffolds do not degrade in the human body within the first 14 days and that their mechanical properties remain stable without remarkable changes. The application of PBS offers a relatively easier interpretation of results. The reduction in the mechanical stability observable via a drop decrease in both the elastic gradient and the plateau stress within the first 7 days and its maintaining at the same level over the next 7 days was accompanied by the conclusion that collagen scaffolds degrade in the human body gradually and that this process is accompanied by a drop loss in their mechanical stability within the first seven days, in which case a further decrease is not anticipated. Thus, the influence of PBS in terms of the simulated body environment is very difficult to compare to the situation observed in the cases of SBF and blood plasma.

Although some studies have focused on the comparison of the impact of various simulated body fluids on protein-based scaffolds [42], to the best of our knowledge, no such study that focuses on collagen scaffolds has been conducted to date. Thus, such information must be determined from individual studies that typically concern scaffolds with various compositions under differing conditions (e.g., media, time exposure, temperature, enzymatic degradation). The comparison of such studies is very complicated and, moreover, the results are often used for the extrapolation of scaffold behavior in vivo, which may lead to inaccurate results. Indeed, the application of alternative media alone is sufficient to result in differing behavior.

A small number of studies focusing on the degradation of materials in simulated body fluids have been presented that take into account the above-mentioned limitations. Tan et al. observed the more rapid degradation of a bone composite scaffold (nano-hydroxyapatite/collagen particles in an alginate hydrogel carrier) in SBF than in deionized water, as proved by the degradation of the alginate molecules. The compressive modulus and shear stress were found to have been significantly reduced in the SBF. The dry weight loss was higher in the SBF, and no weight gain was observed as it was in our study. However, the in vivo evaluation did not determine any obvious degradation even after 8 weeks [43]. Grover et al. [44] optimized collagen and gelatin scaffold properties via the modification of the composition and the cross-linking process. A cross-linked porous collagen scaffold exhibited a ≈5–15% mass loss after 2 weeks in PBS, and the compressive modulus decreased from ≈ 3 to ≈2.2 kPa, which was substantially less than in our study. Zulkifli et al. [45] evaluated the degradation behavior of an HEC/PVA (hydroxyethyl cellulose/poly(vinyl) alcohol) and an HEC/PVA/collagen scaffold in PBS and DMEM and determined differences in terms of weight loss according to the type of scaffold and the medium; more rapid degradation was observed in the PBS. Interestingly, the HEC/PVA presented different values, while the HEC/PVA/collagen presented similar values in both media. The tensile stress differed with respect to each of the specimen–media combinations. Muthukumar et al. [46] evaluated in vitro collagen/chitosan scaffolds for bone regeneration purposes. The testing of degradation in the SBF revealed a weight loss of ≈5–25%. Li et al. [47] studied the degradation of a porous HAP/collagen/PLLA scaffold in PBS. The compressive strength of the specimens without chitin fiber reinforcement gradually decreased over 4 weeks from ≈1.5 to ≈0 MPa.

Various organic molecules (proteins and TRIS) and ions (Na^+^, Cl^−^ and HPO_4_^2−^) are capable of penetrating into the structure of collagen, where they are able to precipitate or crystallize, as demonstrated by the FTIR and XRD, respectively. The penetration of the various molecules and ions into the structure of the collagen may have been influenced significantly by their size and the internal structure of the collagen scaffold. As demonstrated by the FTIR analysis, the final concentration of organic molecules or inorganic ions may be inhomogeneous at different sites in the collagen scaffold; nevertheless, this study demonstrated that the various media employed did not significantly alter the secondary structure of the collagen. The non-uniform penetration of the components into the collagen scaffold that resulted in inhomogeneous mineral precipitates may indicate differing collagen behavior in terms of weight loss, swelling, and mechanical properties in the various media. The discrepancies between the final conclusions of the various researchers may have been due to the use of different simulated body fluids and operating procedures. Since simulated body fluids play a critical role in this process, their compositional aspects must be taken into account in order to avoid the drawing of erroneous conclusions and generalizations.

With respect to our study, SBF treatment led to a significant decrease in the mechanical properties while, conversely, Al-Munajjed et al. [48] observed the increased stiffness (4×) of the collagen scaffold following SBF cultivation as a result of the adsorption of HA on the collagen fibers. Moreover, an HA layer was detected via micro-CT. Lickorish et al. [49] determined the thickness of an HA layer of 2–3 µm following 2 weeks of cultivation in SBF. While such a layer may increase the degree of X-ray contrast, it was not sufficient to be detected by the micro-CT setting used in our study. Interestingly, a study by Permyakova et al. [50] reported an HA layer with a thickness of 100–200 nm on PCL fibers following immersion in SBF for 2 weeks. Zhao et al. [51] reported a layer of HA crystals covering collagen fibers with a thickness of from 20 to 50 nm. While Ficai et al. [52] reported lower porosity following SBF cultivation, this was not observed in our study.

Micro-CT, which offers a number of benefits when compared to SEM, e.g., whole specimen (i.e., the selected volume of interest) 3D analysis that is orientation independent, non-destructive, and time efficient was employed for the evaluation of the 3D structures of the studied specimens. On the other hand, the resolution of micro-CT is more limited than that of SEM; moreover, SEM is still most frequently used for scaffold characterization purposes. The comparison of SEM and micro-CT results for porous materials is not straightforward, as presented in one of our previous studies that focused on the evaluation of pore size [53]. We determined that SEM image analysis resulted in higher pore size values (≈2–3 times) than did micro-CT.

Since significant differences were observed in terms of the mechanical properties and mass loss, we anticipated structural changes as a result of the cultivation procedure. However, the micro-CT analysis revealed only minor changes in the specimens prior to and following cultivation. Moreover, the differences between the cultivation media were even smaller, thus indicating that most of the structural alterations were below micro-CT detection limits. We can assume that the material that adsorbed on the pore walls and into the scaffold structure did not form a thick homogeneous layer (which was also not evident from the SEM images) and presented a generally low X-ray. Adsorption may have been responsible for the minor increase in the scaffold volume and pore wall thickness, as presented in Table 1. The changes in the pore size were negligible. If one compares the pore size (e.g., of 120 µm) with the typical pore wall thickness (8–12 µm; without pore struts), even a 100% increase in the wall thickness would lead to just an ≈8% decrease in the pore size.

## 5. Conclusions

The results point to the need for a reliable in vitro model for the prediction of in vivo collagen scaffold degradation as well as the need for the option to correlate the in vitro to the in vivo situation. This study revealed important differences with respect to the mechanical properties and mass loss of collagen scaffolds as a result of immersion in three differing solutions. Conversely, the 3D structure assessed via micro-CT and secondary structure of collagen assessed via FTIR evinced only negligible changes. The interpretation and interpolation of such results to the real situation can be misleading. The advantage of in vitro laboratory experiments is that they avoid conducting animal experiments, while a further important role lies in the biomaterial development process, namely for the comparison of process parameters, etc. Nevertheless, the simplification of their results and interpolation of the in vivo situation should be avoided in the case of a lack of supporting data from the characterization of degradation processes as conducted, ideally, via several different methods.

## Figures and Tables

**Table 1:** Values of the selected parameters (mean, standard deviation, *n* = 20) determined via micro-CT analysis for the specimens prior to exposure (ORIGINAL) and following 14 days of exposure to the media: blood plasma (PLASMA), simulated body fluid (SBF), phosphate buffer saline (PBS).

Parameter	Original	Plasma	SBF	PBS
Percent object volume [%]	17.41 ± 0.67	19.52 ± 2.62	20.28 ± 3.45	19.35 ± 0.89
Object surface density [mm^−1^]	40.89 ± 2.51	43.84 ± 5.68	43.60 ± 7.14	43.58 ± 5.25
Structure thickness [mm]	0.016 ± 0.001	0.017 ± 0.001	0.017 ± 0.001	0.017 ± 0.001
Structure separation [mm]	0.073 ± 0.010	0.072 ± 0.015	0.073 ± 0.019	0.077 ± 0.025
Open porosity [%]	82.59 ± 0.67	80.48 ± 2.62	79.72 ± 3.45	80.65 ± 0.89

## Supplementary Materials

The following are available online at https://www.mdpi.com/article/10.3390/ma14164388/s1, Figure S1: The stress-strain curves from the compression testing of the samples prior to and after exposure (7 and 14 days) to human blood plasma, SBF and PBS. One sample was selected from each group for each of the graphs. Please note that on the 14^th^ day it was not possible to measure any of the samples exposed to SBF since all the samples had completely degraded with regard both to the elastic gradient and the plateau stress. The arithmetical mean of the stresses between 20% and 30% of the compressive strain was used for the calculation of the plateau stress. The calculation of the elastic gradient was performed via elastic loading and unloading between stresses of 70% and 20% of the plateau stress and the determination of the gradient of the elastic straight lines (graphs on the right), Figure S2: The deconvoluted spectra of the amide I spectral region of the samples (*n* = 10) prior to exposure to the media (ORIG), Figure S3: The deconvoluted spectra of the amide I spectral region of the samples (*n* = 10) following exposure (14 days) to the blood plasma (PLASMA), Figure S4: The deconvoluted spectra of the amide I spectral region of the samples (*n* = 10) following exposure (14 days) to the simulated body fluid (SBF), Figure S5: The deconvoluted spectra of the amide I spectral region of the samples (*n* = 10) following exposure (14 days) to the phosphate buffer saline (PBS).
