# Supplementary material for: Various Simulated Body Fluids Lead to Significant Differences in Collagen Tissue Engineering Scaffolds
Source: Materials (Basel). 2021 Aug 5;14(16):4388. doi: 10.3390/ma14164388 (PMC8399520; doi:10.3390/ma14164388)
Supplement: Supplementary file 1 [file materials-14-04388-s001.zip › materials-1300817-supplementary.pdf]

materials

Supplementary Materials

Various Simulated Body Fluids Lead to Significant Differences in Collagen Tissue Engineering Scaffolds

Tomáš Suchý [1,2,*], Martin Bartoš [3,4], Radek Sedláček [2], Monika Šupová [1], Margit Žaloudková [1], Grażyna Simha Martynková [5] and René Foltán [3]

[1] Department of Composites and Carbon Materials, Institute of Rock Structure and Mechanics, Czech Academy of Sciences, 182 09 Prague 8, Czech Republic; supova@irsm.cas.cz (M.Š.); zaloudkova@irsm.cas.cz (M.Ž.)
[2] Faculty of Mechanical Engineering, Czech Technical University in Prague, 160 00 Prague 6, Czech Republic; radek.sedlacek@fs.cvut.cz
[3] Institute of Dental Medicine, First Faculty of Medicine, Charles University and General University Hospital in Prague, 120 00 Prague 2, Czech Republic; martin.bartos@lf1.cuni.cz (M.B.); rene.foltan@vfn.cz (R.F.)
[4] Institute of Anatomy, First Faculty of Medicine, Charles University, 120 00 Prague 2, Czech Republic
[5] Nanotechnology Centre, CEET, VŠB-Technical University of Ostrava, 708 00 Ostrava-Poruba, Czech Republic; grazyna.simha@vsb.cz
* Correspondence: suchy@irsm.cas.cz; Tel.: +420-777-608-280

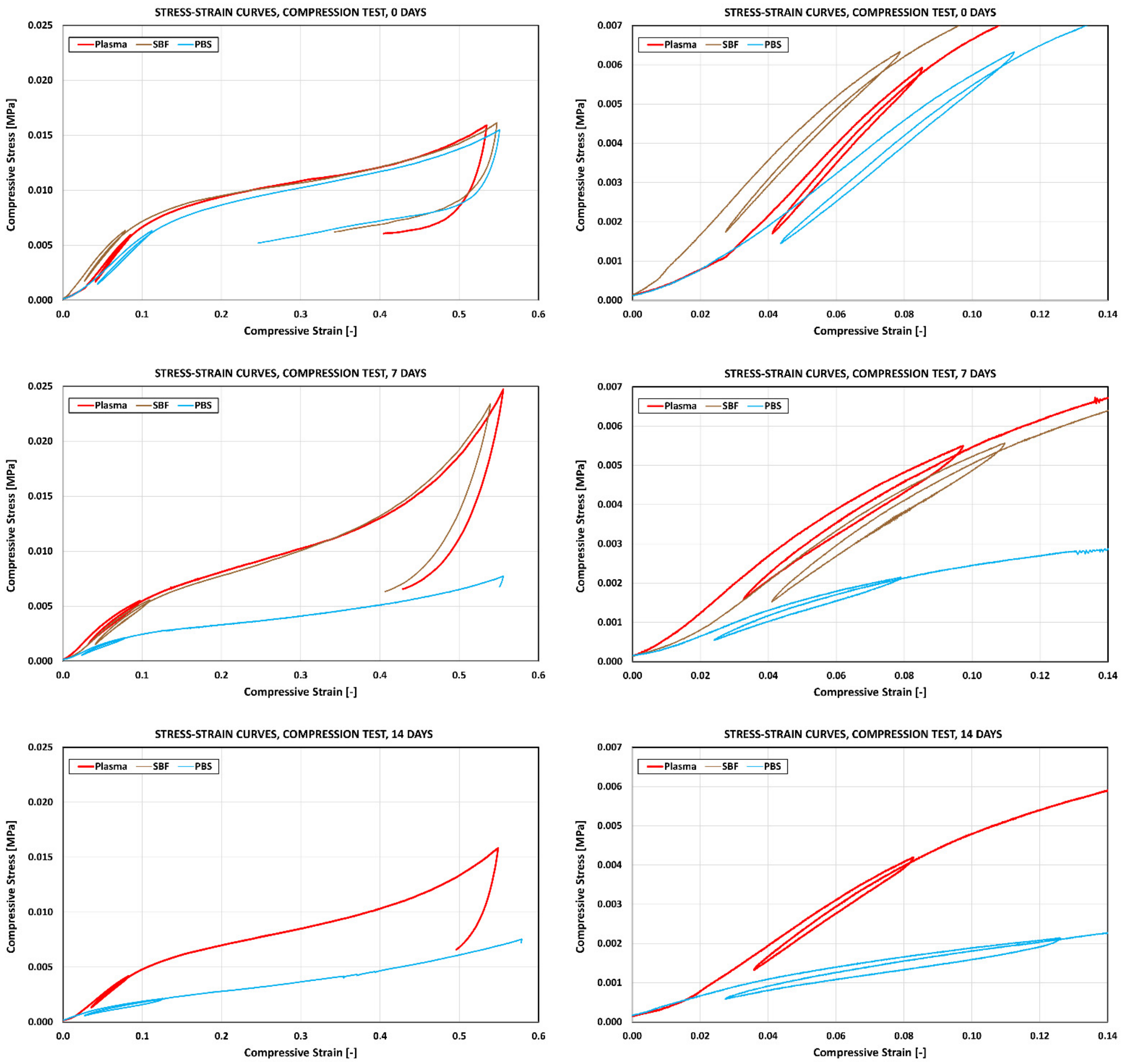

Figure S1. The stress-strain curves from the compression testing of the samples prior to and after exposure (7 and 14 days) to human blood plasma, SBF and PBS. One sample was selected from each group for each of the graphs. Please note that on the 14th day it was not possible to measure any of the samples exposed to SBF since all the samples had completely degraded with regard both to the elastic gradient and the plateau stress. The arithmetical mean of the stresses between 20% and 30% of the compressive strain was used for the calculation of the plateau stress. The calculation of the elastic gradient was performed via elastic loading and unloading between stresses of 70% and 20% of the plateau stress and the determination of the gradient of the elastic straight lines (graphs on the right).

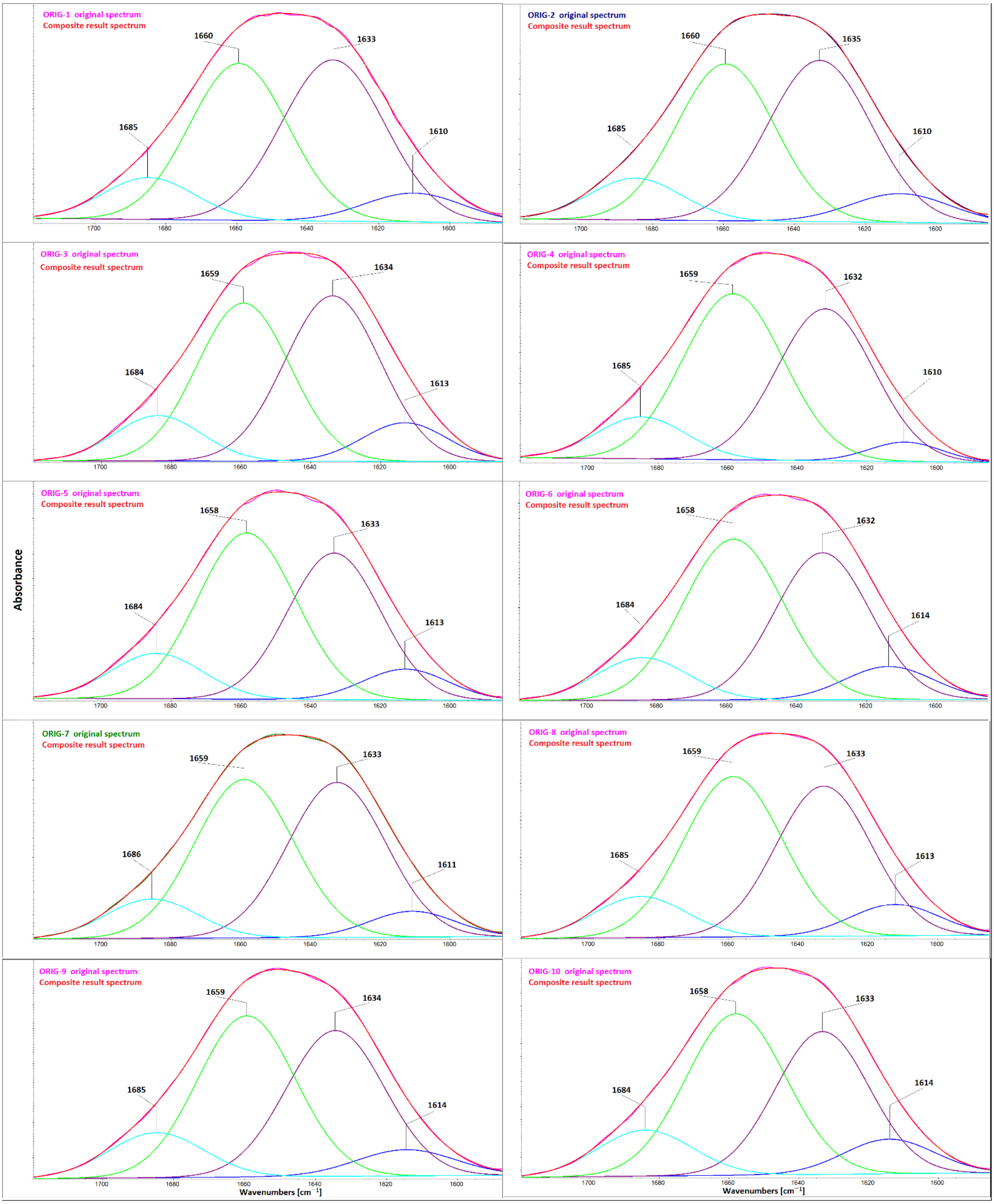

Figure S2. The deconvoluted spectra of the amide I spectral region of the samples (n = 10) prior to exposure to the media (ORIG).

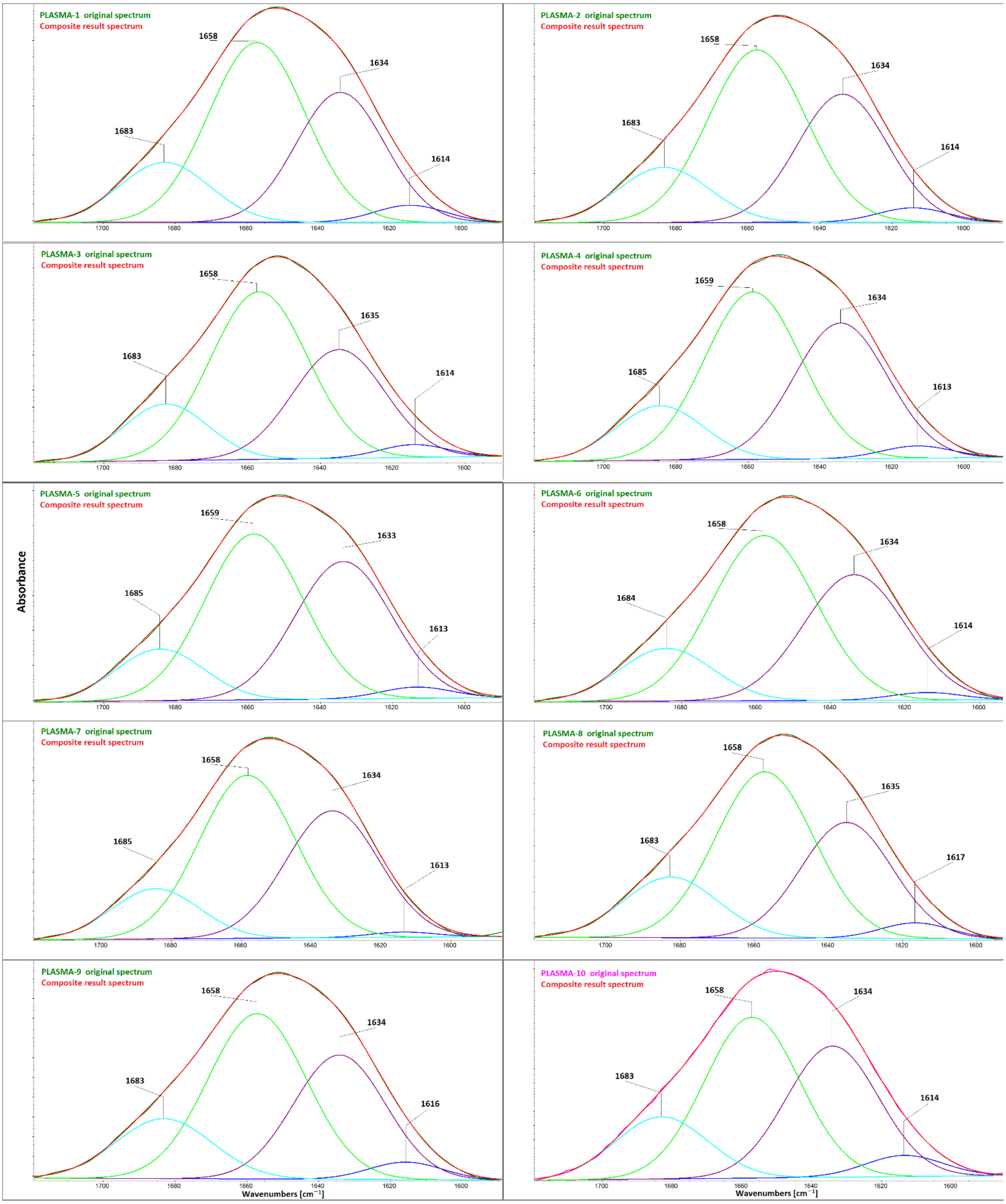

Figure S3. The deconvoluted spectra of the amide I spectral region of the samples (*n* = *10*) following exposure (14 days) to the blood plasma (PLASMA).

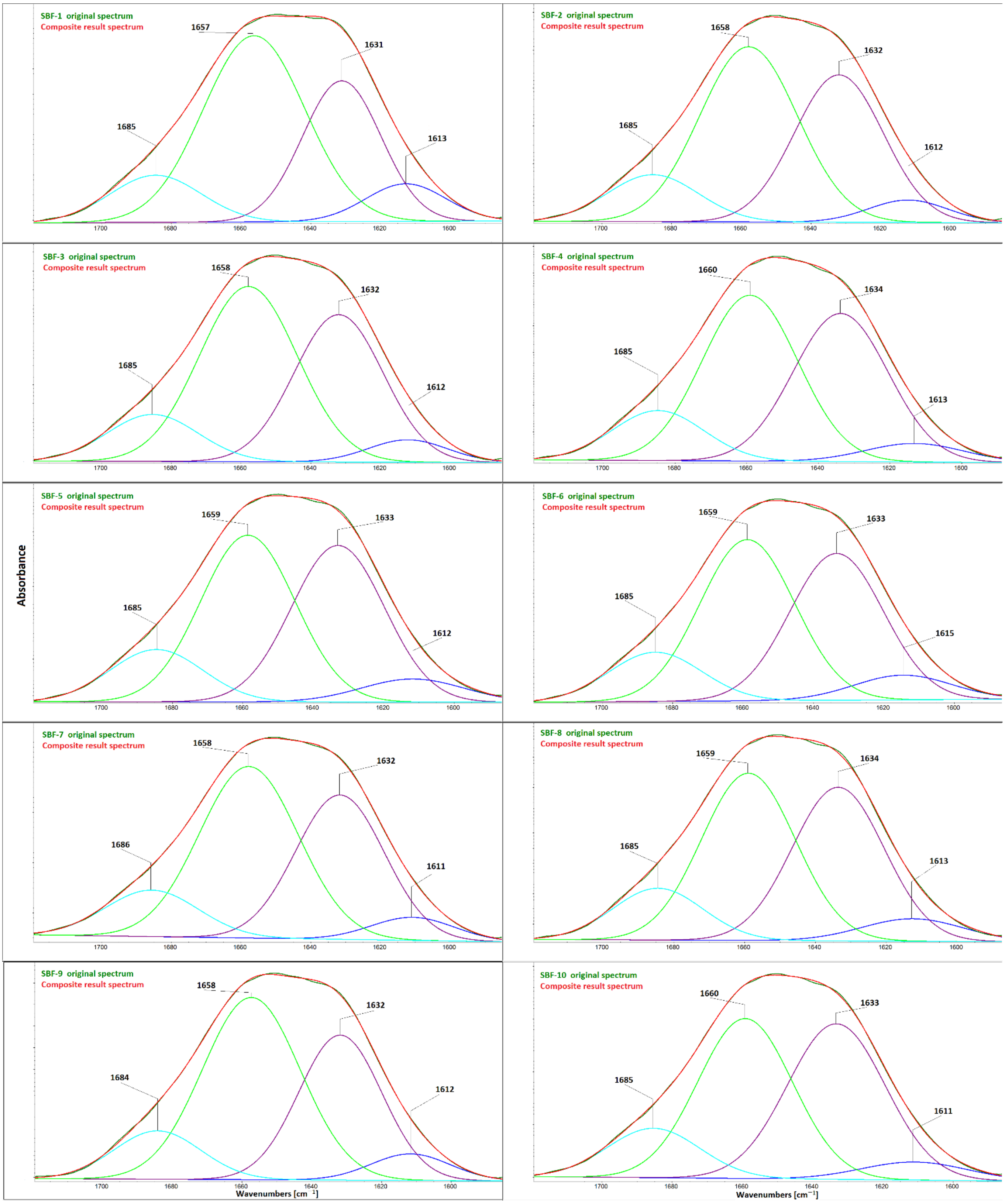

Figure S4. The deconvoluted spectra of the amide I spectral region of the samples (*n* = *10*) following exposure (14 days) to the simulated body fluid (SBF).

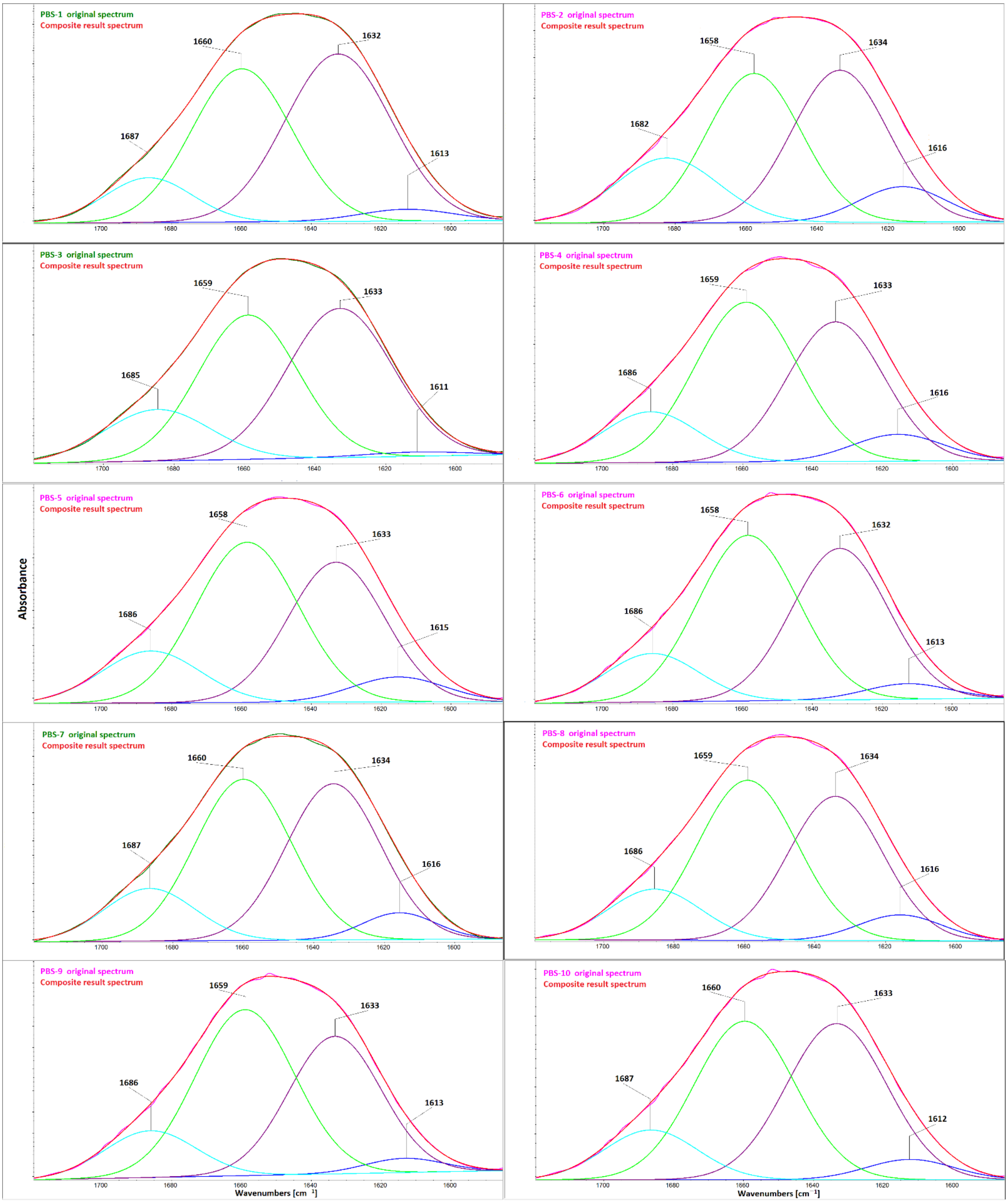

Figure S5. The deconvoluted spectra of the amide I spectral region of the samples (*n* = 10) following exposure (14 days) to the phosphate buffer saline (PBS).
